# Application of circulating tumour cells to predict response to treatment in head and neck cancer

**DOI:** 10.1007/s13402-022-00681-w

**Published:** 2022-06-23

**Authors:** Xi Zhang, Chameera Ekanayake Weeramange, Brett G. M. Hughes, Sarju Vasani, Zhen Yu Liu, Majid Ebrahimi Warkiani, Gunter Hartel, Rahul Ladwa, Jean Paul Thiery, Liz Kenny, Chamindie Punyadeera

**Affiliations:** 1 Saliva and Liquid Biopsy Translational Laboratory, The Centre for Biomedical Technologies, The School of Biomedical Sciences, Faculty of Health, Queensland University of Technology, Kelvin Grove, QLD Australia; 2 Cancer Care Services, Royal Brisbane and Women’s Hospital, Herston, QLD Australia; 3 Faculty of Medicine, University of Queensland, Herston, QLD Australia; 4 Department of Otolaryngology, Royal Brisbane and Women’s Hospital, Herston, QLD Australia; 5 School of Biomedical Engineering, Center for Health Technologies (CHT) & Institute for Biomedical Materials & Devices (IBMD), University of Technology, Sydney, Australia; 6 QIMR Berghofer Medical Research Institute, Brisbane, QLD Australia; 7 Department of medical oncology, Princess Alexandra Hospital, Woolloongabba, Australia; 8 Institute of Molecular and Cell Biology, A*STAR (Agency for Science, Technology and Research), Connexis, Singapore; 9 Saliva & Liquid Biopsy Translational Laboratory, Griffith Institute for Drug Discovery, Griffith University, 46, Don Young Rd, 4111 Nathan, QLD Australia; 10 Translational Research Institute, Woolloongabba, Brisbane, Australia

**Keywords:** Head and neck cancer, Circulating tumour cells, Prognosis, Epithelial-mesenchymal transition, Biomarker, treatment response, Liquid biopsy

## Abstract

**Background:**

Local recurrence and metastasis remain the major causes of death in head and neck cancer (HNC) patients. Circulating tumour cells (CTCs) are shed from primary and metastatic sites into the circulation system and have been reported to play critical roles in the metastasis and recurrence of HNC. Here, we explored the use of CTCs to predict the response to treatment and disease progression in HNC patients.

**Methods:**

Blood samples were collected at diagnosis from HNC patients (n = 119). CTCs were isolated using a spiral microfluidic device and were identified using immunofluorescence staining. Correlation of baseline CTC numbers to 13-week PET-CT data and multidisciplinary team consensus data were conducted.

**Results:**

CTCs were detected in 60/119 (50.4%) of treatment naïve HNC patients at diagnosis. Baseline CTC numbers were higher in stage III vs. stage I-II p16-positive oropharyngeal cancers (OPCs) and other HNCs (*p* = 0.0143 and 0.032, respectively). In addition, we found that baseline CTC numbers may serve as independent predictors of treatment response, even after adjusting for other conventional prognostic factors. CTCs were detected in 10 out of 11 patients exhibiting incomplete treatment responses.

**Conclusions:**

We found that baseline CTC numbers are correlated with treatment response in patients with HNC. The expression level of cell-surface vimentin (CSV) on CTCs was significantly higher in patients with persistent or progressive disease, thus providing additional prognostic information for stratifying the risk at diagnosis in HNC patients. The ability to detect CTCs at diagnosis allows more accurate risk stratification, which in the future may be translated into better patient selection for treatment intensification and/or de-intensification strategies.

**Supplementary information:**

The online version contains supplementary material available at 10.1007/s13402-022-00681-w.

## Introduction

Head and neck cancers (HNC) comprise a range of cancer subtypes arising in the oral cavity, pharynx, larynx, nasal cavity and paranasal sinuses [1, 2]. Approximately 90% of HNCs are squamous cell carcinomas (SCC) [3]. In 2018, it was estimated that there were 887,659 new cases of HNC globally, making it the 7th most common cancer in the world [4]. HNC is also the 7th deadliest cancer worldwide, accounting for an estimated 453,307 deaths in 2018 and a 5-year survival rate of only 40–50% [4]. Smoking and excessive alcohol consumption are classically considered as major risk factors for the development of HNC. However, the epidemiological landscape of HNC has been altered considerably during the last two decades due to a rising incidence of high-risk human papilloma virus (HPV – mainly oropharyngeal cancer sub-type) driven HNC, and a declining incidence of smoking and alcohol-related HNC, especially in the developed world [5, 6].

Even though cancers arising in different sites of the head and neck region are categorized together, these cancers have distinct clinical and biological properties. As such, staging of HNC differs depending on the anatomical site [1]. However, when considering outcomes, HNC can be broadly categorized into two groups as p16 positive oropharyngeal cancer (OPC) and other HNC. It has been reported that p16 positive OPCs (indirect biomarker to evaluate tumour HPV status) exhibit better clinical outcomes compared to other HNCs, including p16 negative OPCs. Thus, the staging system recommended by the American Joint Committee on Cancer (AJCC 8th Edition) for p16 positive OPC is quite different from that of other HNCs [7]. This change was largely based upon lower recurrence and treatment failure rates observed for p16 positive OPC, i.e., approximately 10–20% [1, 8]. Overall, the recurrence and treatment failure rates remain at about 50% for other HNC cases, while the distant metastasis rates range from 4 to 26% [9–12].

In the current clinical workflow, fluorine-18 fluorodeoxyglucose (FDG) positron emission tomography-computed tomography (PET-CT) scanning is used for post-treatment response assessment and characterization of treatment response at 13-weeks [13]. However, despite its simplicity, there are major limitations of using PET-CT scanning to a priori predict treatment response, due to its low positive predictive value caused by post-radiation inflammation and the lack of standardisation of qualitative reporting [14]. Hence, treatment intensification/de-intensification is not possible in the initial stages of treatment. As such, there is an unmet clinical need to identify reliable prognostic biomarkers at diagnosis to customize treatment regimens with the aim to improve patient outcomes [15].

Circulating tumour cells (CTCs) are formed when cells shed from primary or metastatic tumour sites into either the blood stream or the lymphatic system. The presence of pathways that accelerate tumour invasiveness and metastatic properties, designated by high dissemination rates of tumour cells into circulation, is an indicator of tumour aggressiveness. Epithelial-mesenchymal transition (EMT) of CTCs promotes this process [16, 17]. CTCs have been reported to be detected in almost every type of solid tumour and they have been suggested as prognostic indicators for breast cancer [18], prostate cancer [19] and colorectal cancer [20, 21]. However, the rarity of CTCs in circulation poses a significant challenge for their utilization as robust disease-specific biomarkers [22]. Early CTC detection technologies have used labelling methods, either immunocapture, such as the FDA-approved CELLSEARCH® platform [23], or density-based techniques [21]. There remain limitations to the label-based techniques, such as under-expression of markers used to capture CTCs [24]. Novel emerging technologies are utilizing label-free technologies to address this problem. Warkiani et al. and Papautsky et al. developed low-cost, spiral microfluidic devices that are capable of isolating CTCs base on their size and deformability, overcoming the limitations of previous antibody-based isolation strategies [24–26].

We hypothesize that CTCs can be used as prognostic biomarkers to predict responses to treatment at 13 weeks in both p16 positive OPC patients and other HNC patients. The overall objective of the current study was to stratify the risk at diagnosis for response to treatment or disease progression in locoregionally advanced HNC patients based on 13-week PET-CT data and multidisciplinary team (MDT) HNC decision outcomes. Considering that EMT of tumour cells plays an essential role in promoting the migration and invasion potentials of tumour cells, we also evaluated the expression level of cell-surface vimentin (CSV), a mesenchymal marker that is known to be associated with a poor prognosis in cancer patients [27–29].

## Materials and methods

### Study participants

This study complies with the 2013 Declaration of Helsinki [30] and the Australian Code for Responsible Conduct of Research [31]. We obtained ethics approvals from the human research ethics committee of Metro South Health District (approval number: HREC/12/QPAH/381) and Queensland University of Technology (approval number: 1400000617) for this study. Loco-regionally advanced HNC patients who were > 18 years of age and willing to be followed up were included in the study. We excluded HNC patients with salivary gland, thyroid and nasopharynx cancer and patients with metastatic cutaneous malignancies. All study participants provided informed written consent prior to inclusion in this study. Tumours of each patient were staged according to AJCC Cancer Staging Manual 8th Edition [7].

Patients were grouped into p16 positive OPC and other HNCs (oral cavity cancer, p16 negative OPC, hypopharynx cancer, larynx cancer and cancer with unknown primary site) based on their clinical and biological characteristics. Following written informed consent, 15 ml blood was collected from HNC patients in K2E EDTA (Cat#: 45505, Greiner Bio-One, Gloucestershire, UK) vacutainers. All HNC patients were treatment naïve at the time of baseline blood collection. Patient treatment responses were determined and categorised by analysing the clinical reports of FDG-PET-CT imaging and MDT consensus at 13 weeks post-treatment. PET-CT scans were performed and assessed as either “complete metabolic response”, “equivocal” or “positive” by experienced radiologists. Patients were deemed to have a complete metabolic response when their PET-CT scans showed no abnormal focal FDG uptake or only diffuse FDG uptake without a corresponding anatomical abnormality on CT. Patients were deemed to have an incomplete response if they had a positive PET-CT or they died due to HNC before the 13 weeks post-treatment CT scan was performed. A “positive” PET-CT assessment was determined when there was a focal FDG uptake at the sites and a corresponding abnormality of anatomical structure with a greater intensity than that of the background. PET-CT scans that showed reduced focal FDG uptake in comparison to baseline imaging, but still higher than adjacent normal tissues, were classified as equivocal. For patients with equivocal PET-CT scans, a further 6–8 weeks PET-CT scan was performed as well as MDT consensus based on all available imaging and clinical data to determine whether a patient had a complete or an incomplete response.

### Sample size calculations

A comparison of CTC counts between patients with a complete response versus those without has ≥ 90% power with a sample size of 100 patients, assuming that half are responders and that responders have an average CTC count of 1.0 (Poisson rate) and that non-responders have a rate of at least 1.75 using a Poisson-based comparison of rates between the two groups and α level = 0.05.

### Circulating tumour cell isolation and enrichment

To reduce the cellular components passing through the spiral microfluidic chip, an initial red blood cell lysis using RBC lysis buffer (cat# 786 − 649, G-Bioscience, MO, USA) was performed as per our previous publications [32, 33]. Briefly, cells were centrifuged and cell pellets were resuspended in 10 ml sheath buffer (1xPBS, 2 mM EDTA, 0.5% BSA). Next, Tygon® tubing was inserted into the inlet/outlet of the spiral microfluidic chip, and the inlet tubing connected to a syringe pump. The spiral microfluidic chip was positioned and fixed onto a phase contrast microscope to monitor the fluid flow. The outlet tubing was connected to two sterile 15 ml collection tubes. An initial priming run was performed using the sheath buffer at a flow rate of 2.0 ml/min for 5 min. Patient samples were loaded into a 10 ml syringes (Cat# # 51903, Terumo, Tokyo, Japan) and pumped through the spiral microfluidic chip using the syringe pump at a flow rate of 1.7 ml/min. The outputs were collected and spun down at 500× g for 5 min. The enriched cells were then fixed with 4% PFA for 10 min and spun onto a polylysine-coated glass slide (ThermoFisher, MA, USA).

### Immunofluorescent staining of circulating tumour cells

Immunofluorescence (IF) staining was used for the detection of CTCs enriched by the spiral microfluidic chip. Cytospun samples were briefly washed with PBS and air dried. Next, the cells were permeabilised with 0.1% Triton-X100 for 10 min. After washing the samples with 1xPBS, they were blocked with 10% FBS. After washing with PBS, the cells were stained with a cocktail of antibodies. CTCs were identified by IF using anti-cytokeratin monoclonal antibody AE1/AE3 (cat# 41-9003-82, ThermoFisher), anti-CD45 monoclonal antibody (Cat# 340943, BD Biosciences, NJ, USA) and DAPI. Cells were further characterised for CSV expression using monoclonal anti-CSV antibody 84 − 1 (cat# H00007431-MF08, ThermoFisher, USA). The slides were incubated for 1 h at room temperature, washed 3 times in PBS, cover slipped and imaged under a fluorescent microscope (Zeiss Imager Z2, Zeiss, Oberkochen, Germany).

CTCs were defined as cytokeratin-positive, DAPI-positive cells and CD45-negative cells that were larger than 10 μm with intact cell membrane. Cells staining positive for CD45 and DAPI and negative for pan-cytokeratin were determined to be white blood cells and as such excluded from the analysis. FaDu, a HNC cell line expressing cytokeratin was used as a positive control for the immunostaining procedures. FaDu cells were cultured in RPMI-1640 medium (Life Technologies) supplemented with 10% Foetal Bovine Serum (Life Technologies) at 37 °C in a humidified atmosphere containing 5% CO_2_. FaDu cells were harvested when they reached 80% confluency as per our publication [34], and then fixed with 4% PFA for 10 min and spun onto polylysine-coated glass slides. Scanning of the CTC slides was performed on the Zeiss Axio Z2 microscope and sequential images were captured after fluorescent staining. A multi-exposure protocol was used to detect the signals. A tile scanning mode was setup to image the whole surface area of the enriched cells on the glass slides. Zen software was used to interrogate the images and constrained iterative algorithms were used for image deconvolution. Most of the CTC slides were evaluated by two independent researchers.

### Assay performance characteristics for the CTC enumeration

To evaluate the suitability of immunofluorescent staining for enumerating CTCs in HNC patient samples, duplicates of blood samples from 16 HNC patients were processed and analysed independently using the methods described above. Repeatability of the assays was examined using agreement analysis and correlation analysis.

### Statistical analysis

All statistical analyses were performed using GraphPad Prism 8 (GraphPad Software Inc., La Jolla, CA, USA), R (R Development Core Team. Vienna, Austria) and JMP Pro (v16.1 SAS Institute, Cary NC, USA). GraphPad was used to analyse baseline clinical characteristics. Continuous variables were assessed for normality using the Shapiro–Wilk test. If an approximate normal distribution could not be achieved, Kruskal–Wallis tests with Dunn’s multiple comparisons tests were performed to compare multiple groups. CTC counts were modelled as Poisson variables using generalised linear models with a Poisson distribution to generate 95% confidence intervals for the mean counts (i.e. Poisson Rates) and to provide comparisons between groups. Logistic regression was used to model treatment response versus clinical and biomarker variables. ROC curves were used to depict the predictive power of the model and AUC, sensitivity and specificity were provided with 95% confidence intervals. Cut-offs for sensitivity and specificity were chosen to maximize Youden’s index. Multivariable logistic models used LASSO penalised regression with leave one out cross validation to find a parsimonious predictive model. The initial model included baseline CTC numbers and baseline clinical parameters: cancer type, age, gender, alcohol consumption habit, N stage, tumour stage and size and number of nodes, and used LASSO to select a subset of predictors to predict the treatment outcome at 13 weeks.

## Results

### Participant characteristics

**Fig. 1:**
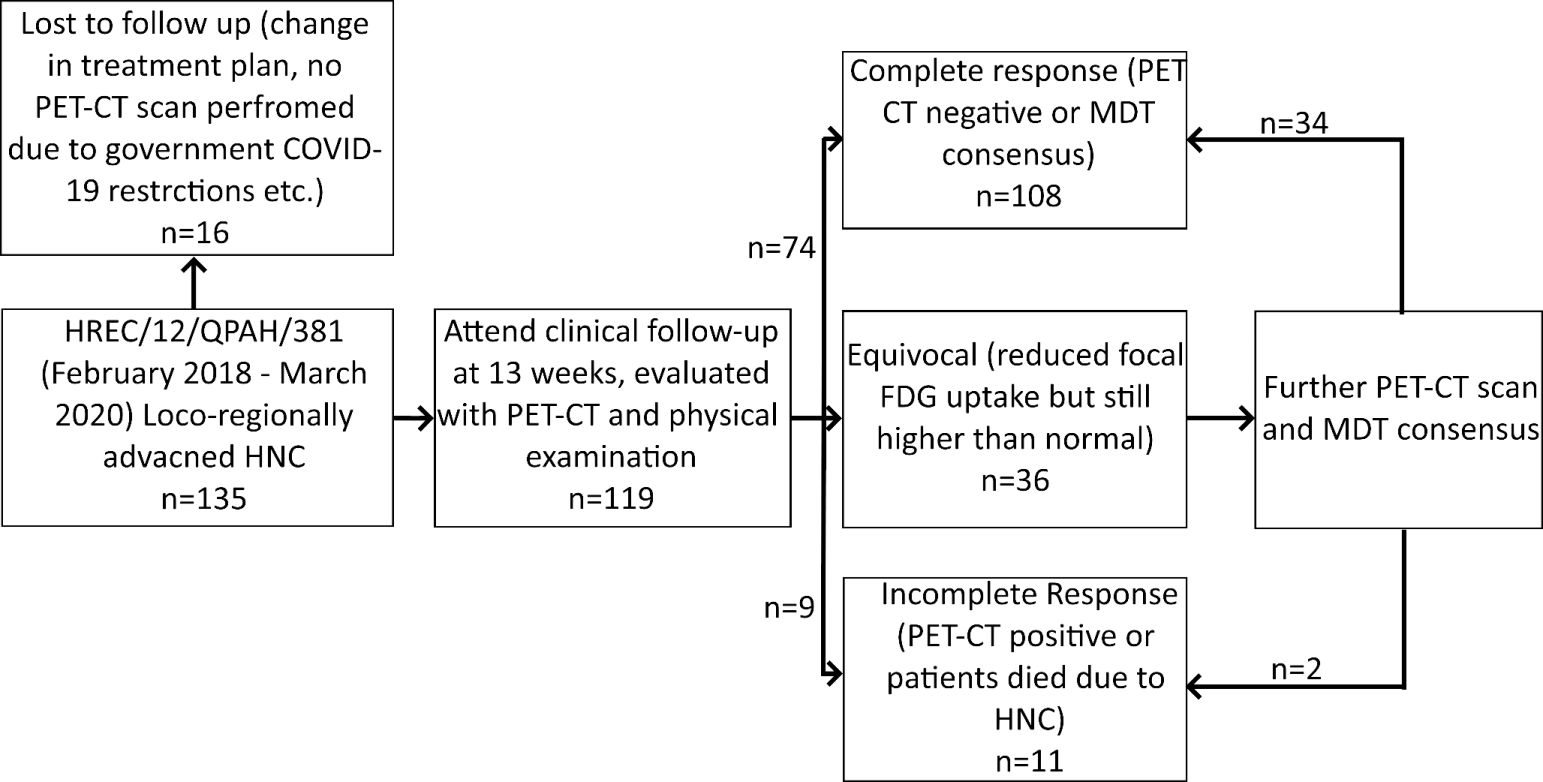
Study flow chart

In total, 135 HNC mucosal, locoregionally advanced SCC patients were recruited to this study (Fig. 1). Out of these 135 patients, 16 did not undergo PET/CT scans at 13 weeks for several reasons (e.g. COVID-19 related access issues, failure to attend clinical appointments or having surgery as their principle modality of treatment) and, as such, were excluded from the analysis. The clinical characteristics of the remaining participants (n = 119) are listed in Table 1. During their PET-CT scan at 13 weeks post-treatment, 74 patients were categorised to have a complete response, 9 patients to have an incomplete response, and 36 patients to have an equivocal response. Further PET-CT scan and MDT consensus categorised these patients as having a complete response (n = 34) or an incomplete response (n = 2). In total, 108 out of 119 (90.8%) patients achieved a complete response post chemoradiotherapy. Incomplete responses were observed in 11 patients (9.2%).

**Table 1:** Clinicopathological summary for mucosal head and neck patient cohort

Patients	Complete Response	Incomplete Response
n = 119	108	11
Gender		
Male	89 (82.4%)	9 (81.8%))
Female	19 (17.6%)	2 (18.2%)
< 60	59 (54.6%)	3 (27.3%)
≥ 60	49 (45.4%)	8 (72.7%)
Cancer location		
Oral Cavity	14 (13.0%)	7 (63.6%)
Oropharynx	88 (81.5%)	3 (27.3%)
Hypopharynx	1 (0.9%)	0 (0%)
Larynx	3 (2.8%)	1 (9.1%)
Unknown	2 (1.8%)	0 (0%)
Tumour stage (AJCC 8th Ed)		
I	44 (40.7%)	3 (27.3%)
II	33 (30.6%)	1 (9.1%)
III	22 (20.4%)	3 (27.3%)
IVa or IVb	9 (8.3%)	4 (36.4%)
Tumour p16 status		
Positive	90 (83.3%)	3 (27.3%)
Negative	10 (9.3%)	6 (54.5%)
no data	8 (7.4%)	2 (18.2%)
CTC status		
CTC positive	50 (46.3%)	10 (90.9%)
CTC negative	58 (53.7%)	1 (9.1%)

### Immunofluorescent characterisation of CTCs

CTCs were successfully enriched using the spiral microfluidic device. To optimise the immunofluorescent staining protocol, human HNC FaDu cells were immobilised onto polylysine-coated glass slides, after which immunofluorescent staining was performed (Supplementary Fig. 1). As expected, the FaDu cells reacted specifically with the fluorescent anti pan-cytokeratin antibody but not the anti-CD45 antibody. CTCs were visualised using immunofluorescence staining (Supplementary Fig. 2).

### Repeatability of CTC enumeration

The repeatability of the CTC enumeration using immunostaining is summarised in Supplementary Fig. 3. By analysing duplicate samples from 16 HNC patients, we evaluated the agreement between the two measurements using a contingency test (Supplementary Fig. 3 A). The test showed an agreement statistic Kappa of 0.87, which is considered perfect agreement. We also evaluated the correlation between the two measurements (Supplementary Fig. 3B) and found significant correlations (Pearson correlation coefficients *r* = 0.9708, *p* < 0.0001),

### CTC enumeration in HNC blood samples at diagnosis

**Fig. 2:**
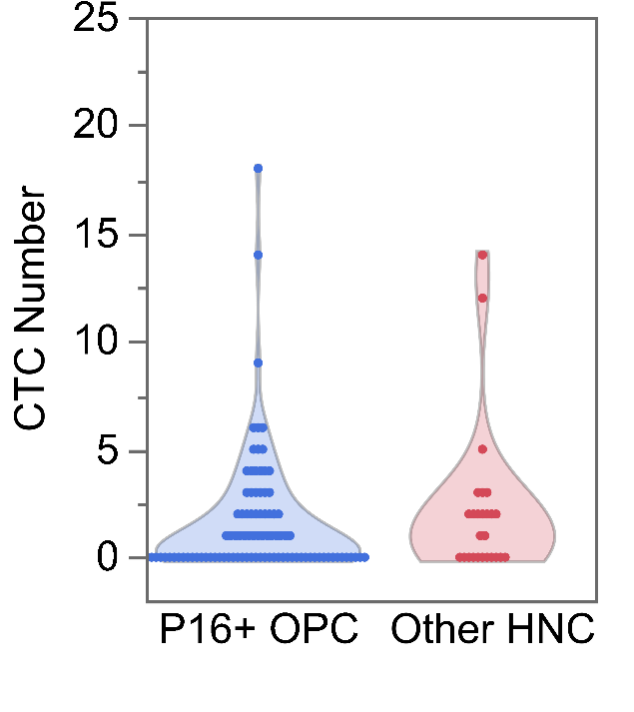
Enumeration of circulating tumour cells (CTCs) in p16 positive oropharyngeal cancer patients and other head and neck cancer patients

We observed no differences in the number of CTCs between p16 positive OPC compared with other HNCs. We detected at least 1 CTC in 3 ml blood samples from 60 HNC patients (50.4%). Noticeably, in HNC patients with an incomplete response, we found at least 1 CTC in 10 out of the 11 patients (90.9%). We also investigated whether tumour p16 status affects enumerated CTCs in patient blood samples. We found that patients with p16-positive OPC had a slightly lower number of CTC counts (mean 1.61 95% CI 1.37, 1.89) compared to patients with other HNCs (2.15 95% CI 1.65, 2.81), but this difference was not statistically significant (*p* = 0.061, Fig. 2).

**Fig. 3:**
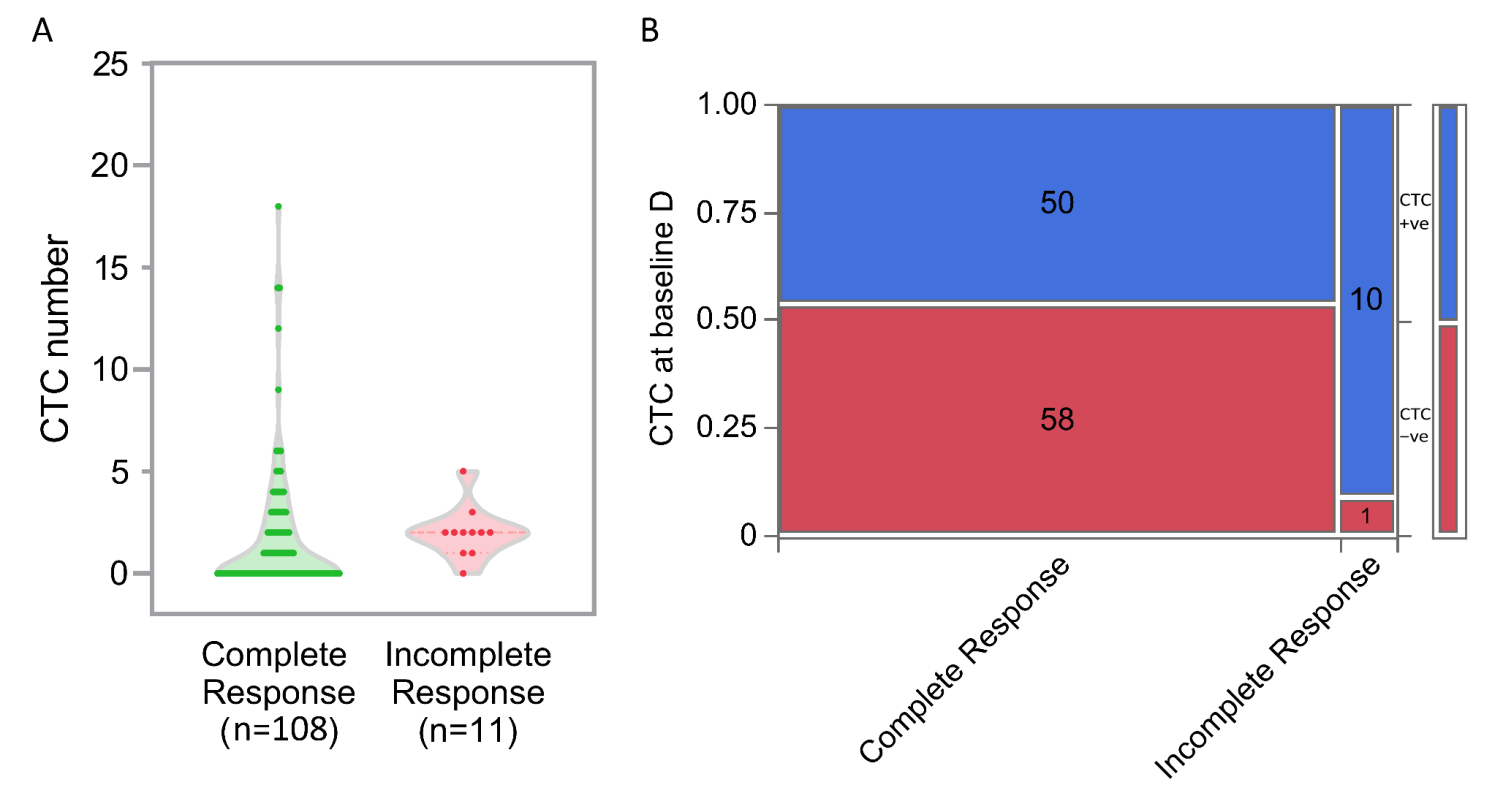
(**A**) Circulating tumour cell (CTC) numbers in all HNC patients (n = 119) with a complete response and an incomplete response. (**B**) Contingency analysis of baseline CTC circulating tumour cells by 13 weeks related to treatment response status

Among the patients with a complete response at 13 weeks post- treatment, the CTC numbers in the blood samples ranged from 0 to 18 with a mean of 1.58 (Poisson rate, 95% CI 1.34, 1.86), whereas in those with an incomplete response, the CTC numbers ranged from 0 to 5 with a mean of 2.33 (95% CI 1.10. 4.94) (Fig. 3A). When comparing HNC patients with a complete response, 46.3% had baseline CTCs and 53.7% had no baseline CTCs (χ² *p* = 0.0048). Among patients with no CTCs at baseline, 98.3% achieved a complete response at 13 weeks, and only 1.7% had an incomplete response.

### Baseline CTCs are found in p16-positive OPC patients with an incomplete treatment response

**Fig. 4:**
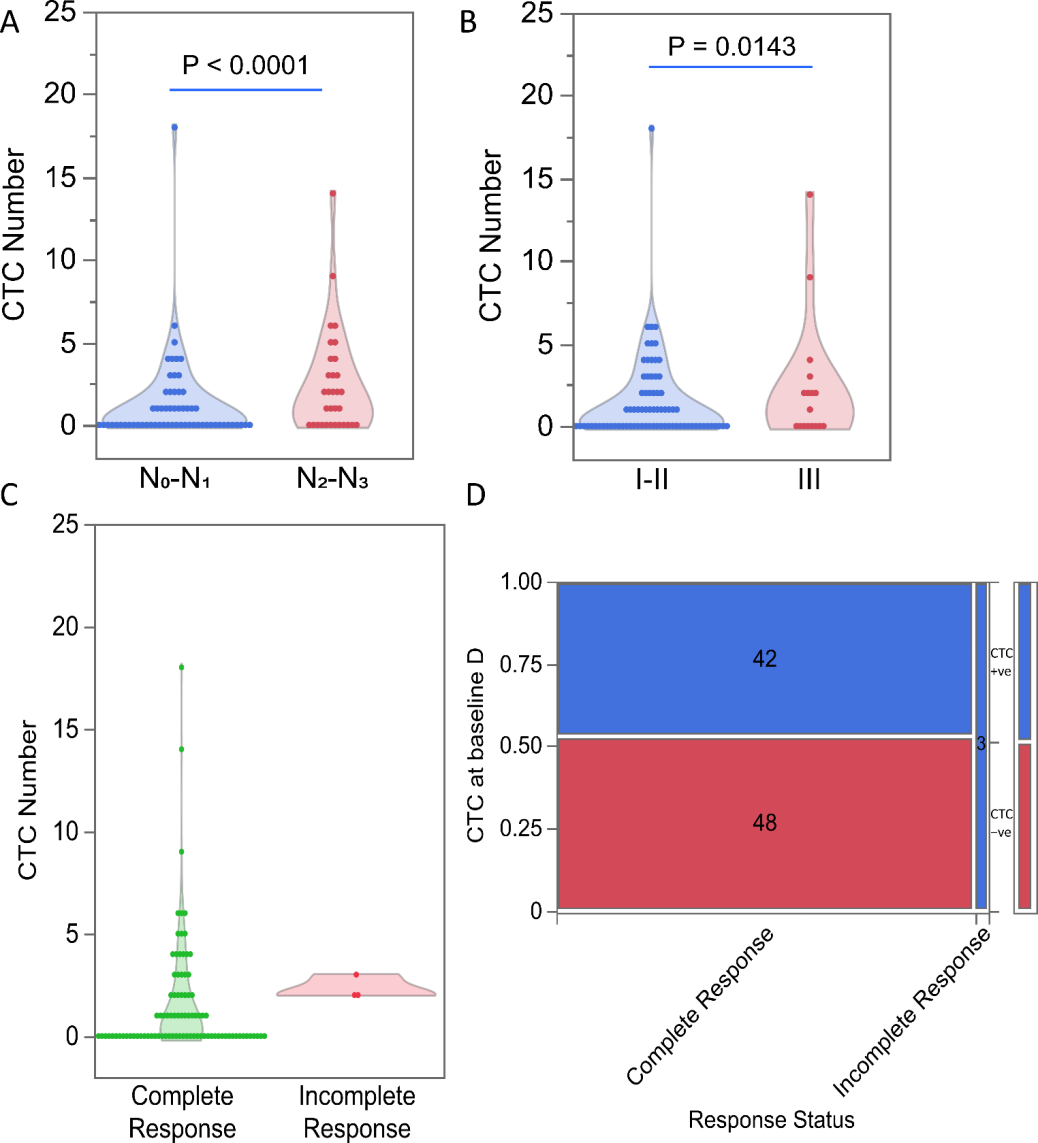
Enumeration of circulating tumour cells (CTCs) in p16-positive oropharyngeal cancer (OPC) patients categorised by (**A**) N stage, (**B**) tumour stage and (**C**) treatment response. (**D**) Contingency analysis of baseline CTCs by treatment response status in p16-positive OPC patients

We found that in p16-positive OPC patients with N stage 0 or 1, CTC numbers ranged from 0 to 18 with a mean of 1.21 (Poisson 95% CI 0.96, 1.52) and that in patients with N stage 2 and 3, CTCs numbers ranged from 0 to 14 with a mean of 2.38 (Poisson 95% CI 1.89, 2.98, Fig. 4A). The CTC numbers were significantly higher in patients with TNM stage 2 and 3 compared to patients with TNM stage 0 or 1 (*p* < 0.0001). The CTC numbers were higher in patients with stage III tumours (range 0–14, Poisson rate 2.29, 95%CI 1.67, 3.15) than in patients with stage I or II tumours (range 0–18, Poisson rate 1.45, 95% CI 1.21, 1.75) (Fig. 4B). These observations indicate that the CTC numbers in patient blood samples increase with more advanced tumour stages (*p* = 0.0143). Mean CTC numbers were significantly higher in p16-positive OPC patients who exhibited incomplete responses compared to patients with complete responses (Fig. 4C). More importantly, CTCs were detected in all 3 patients with an incomplete response, while only 42/90 patients with a complete response had CTCs in their baseline samples (χ² *p* = 0.0346, Fig. 4D).

### CTCs in other HNC patients with an incomplete treatment response

**Fig. 5:**
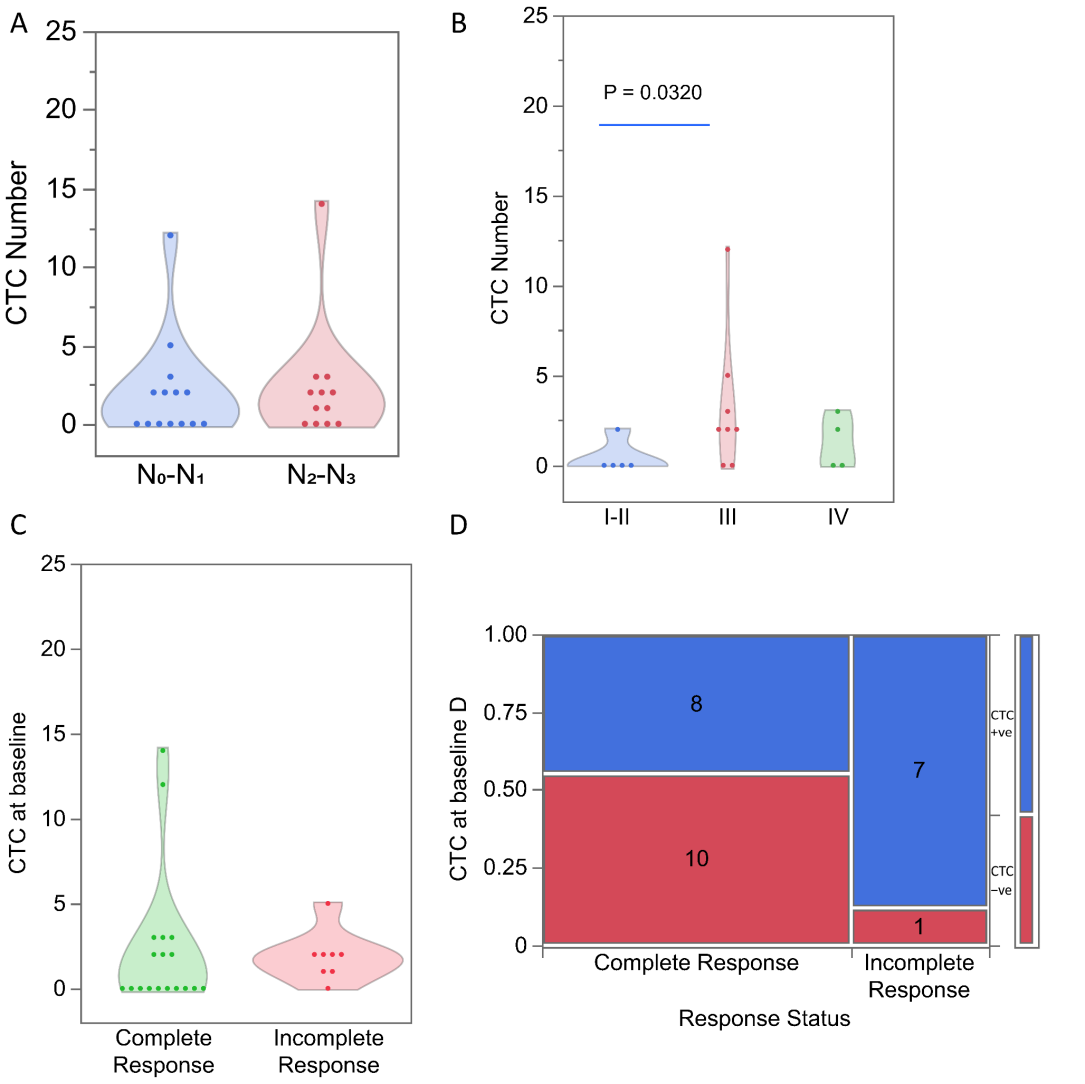
Enumeration of circulating tumour cell (CTC) counts in other head and neck cancer patients by (A) N stage, (**B**) tumour stage and (**C**) treatment response. (**D**) Contingency analysis of baseline CTC by treatment response status

We found that in patients with other HNCs with N stages 0 and 1, the CTC number ranged from 0 to 12 with a mean of 2.0 (Poisson 95% CI 1.35, 2.95) and that in patients with N stage 2 and 3, the CTC numbers ranged from 0 to 14 with a mean of 2.33 (Poisson 95% CI 1.58, 3.45, Fig. 5A). The CTC numbers in patients with other HNCs were higher in patients with more advanced tumour stages (Fig. 5B). A Poisson-based comparison of the 3 groups of patients showed a significant difference (*p* = 0.0043) in CTC numbers. Patients with stage I or II tumours had CTC numbers between 0 and 2 (mean 0.40, 95% CI 0.09, 1.82), whereas in patients with tumour stage III the CTC numbers ranged from 0 to 12 (mean 3.25, 95%CI 2.13, 4.95) and in patients with tumour stages IVa and IVb the CTC numbers ranged from 0 to 3 (mean 1.25, 95%CI 0.48, 3.26). Patients with stage III other HNCs exhibited significantly higher CTC counts compared to patients with stage I and II (*p* = 0.032), whereas no significant differences were observed compared to patients with stage IVa and IVb (*p* = 0.160). Furthermore, in this cohort no significant difference in CTC counts was observed between patients with a complete response (range from 0 to 14, mean 2.28, 95%CI 1.65, 3.14) or an incomplete response (range from 0 to 5, mean 1.88, 95%CI 1.10, 3.10, Fig. 5C). At least one CTC was detected at baseline in 8/18 complete responders and in 7/8 HNC patients with an incomplete response (χ² *p* = 0.0403, Fig. 5D).

### CSV expression on circulating tumour cells is higher in patients with an incomplete treatment response

**Fig. 6:**
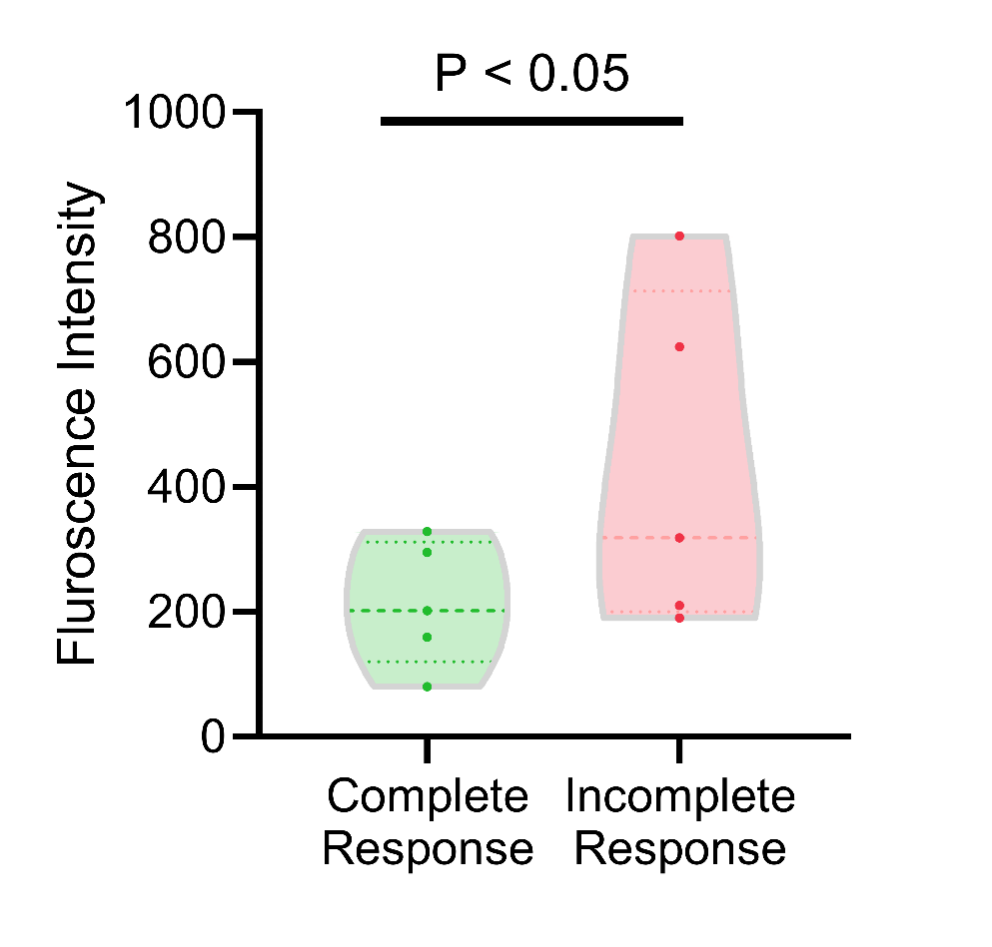
Cell surface vimentin expression on circulating tumour cells (CTC) isolated from locoregionally advanced head and neck cancer patients who had a complete treatment response versus those who had an incomplete response

We also evaluated CTC samples from 10 patients for CSV expression levels (Fig. 6). Significantly higher (*p* < 0.05) CSV expression levels were found in baseline CTCs in patients who had an incomplete response (n = 5) at 13 weeks compared to patients with a complete response (n = 5).

### Presence of CTCs at baseline serves as an independent predictor of treatment response at 13 weeks

**Fig. 7:**
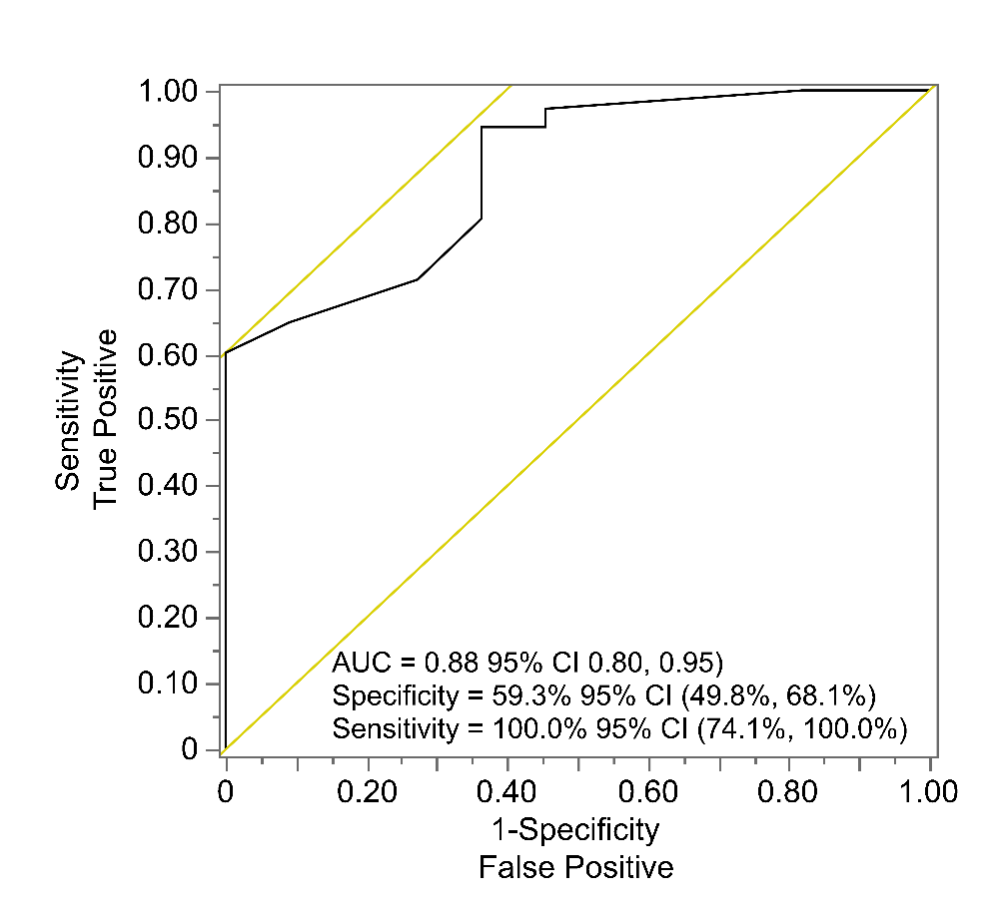
Receiver Operative Characteristic (ROC) curve evaluation of the differentiating power of CTC numbers comparing head and neck cancer patients with a complete response with those with an incomplete response

We have applied a logistic regression model using CTC presence at baseline to predict patients’ treatment response at 13 weeks (Fig. 7). We found that CTCs at baseline are associated with response at 13 weeks post treatment (*p* = 0.0005). To assess the effect of including clinical parameters in the predictive model, we used LASSO Logistic regression with leave-one-out validation to combine baseline CTC number with other available predictors (cancer type, age, gender, alcohol consumption habit, N stage, tumour stage and size and number of nodes), to predict the treatment outcome (complete response versus incomplete response) of the patients at 13 weeks post treatment. The LASSO model eliminated all clinical parameters and did not improve the model beyond CTC counts at baseline and cancer type. The area under the ROC curve for this model was 0.88 (95% CI 0.80, 0.95) with a sensitivity of 100% (95% CI 74.1%, 100%) and a specificity of 59.3% (95% CI 49.8%, 68.1%).

## Discussion

Despite advances that have been made in multimodality treatment strategies, HNC remains one of the most debilitating cancer types, largely due to its high recurrence and metastasis rates [35, 36]. Metastasis occurs when tumour cells break away from the primary tumour and enter either into the lymphatic system and/or the circulation, a phenomenon that is not always clinically or radiographically evident in its initial stages. Detection of CTCs provides an alternative approach to identify tumours with a greater metastatic potential. As such, CTCs can be considered as indicators of tumour aggressiveness which may predict response to treatment in patients. Hence, CTCs can be used as a minimally invasive, real-time tool to triage patients requiring intensive treatment and frequent monitoring.

The prognostic potential of CTCs has been shown across many cancer types, including breast, prostate, colorectal cancer and HNC [24, 37–43]. However, due to technological dissimilarities in CTC isolation and characterization, a general consensus has not yet been reached regarding their clinical utility as prognostic indicators for managing HNC patients. Fortunately, recent advances in label-free, microfluidic-based CTC isolation and enrichment technologies have revolutionized the field of CTC application. These technologies are simple, cost-effective and can efficiently capture CTCs based on their size and deformability [24–26]. Despite these advances, however, CTC technologies are yet to be translated into clinical practice. To address this knowledge and clinical gap, we set out to investigate the prognostic utility of CTCs in a large cohort of HNC patients using a protocol that is amenable to current clinical practice.

We found that absence of CTCs at diagnosis is positively correlated with treatment outcomes in HNC patients. At least 1 baseline CTC was detected in 50.04% of HNC patients and in 90.9% (10/11) of HNC patients with an incomplete response based on 13th week PET-CT scan results and MDT decision making. Furthermore, the mean baseline CTC count was found to be significantly higher in HNC patients with an incomplete response compared to that in patients with a complete response (2.33 vs. 1.58). Among 11 patients who had an incomplete treatment response, 3 (3.2%) carried p16 positive OPCs and 8 (30.8%) other HNCs. Our data confirm previous reports [44–46] that p16 positive OPCs have a better prognosis. All three of p16 positive OPC patients who did not respond to treatment had CTCs at baseline, while 7/8 patients with other HNCs had CTCs detected at baseline. A regression model considering CTC counts at baseline and cancer type was able to predict treatment outcomes with up to 100% (74.1 − 100%) sensitivity and 59.3% (49.8%, 68.1%) specificity (AUC 0.88).

Several previous studies, including ours, have reported that baseline CTC positivity may be associated with a poor prognosis in HNC patients. Kulasinghe et al. [24] reported that CTCs were detected in 47.8% of 23 HNC patients (Stages I-IV) at baseline using microfluidic-based CTC enrichment (ClearCell® FX1) and that CTC positivity was associated with progression-free survival. However, this latter study used a heterogenous group of HNC patients. In contrast, our current study used p16 positive OPCs and other HNCs with CTCs being used as independent predictors of progression. This finding may have important implications for stratifying HNC patients at diagnosis for the risk of treatment failure based on CTC numbers. Furthermore, Inhestern et al. [47] reported that high CTC counts at baseline are predictive of recurrence risk. They used The Maintrac CTC analysis in their study and detected CTCs in 80% of HNC patients prior to treatment [47]. Conversely, Tinhofer et al. (2014) reported that the presence of CTCs was not indicative of neither overall survival nor disease-free survival [48]. They used nested PCR amplification of EGFR transcripts as an indicator for CTCs and detected these in 29% of HNC patients at baseline according to their criterion [48]. However, a recent meta-analysis involving 1,054 HNC patients indicated that the presence of CTCs is indicative of a poor prognosis [49]. Our current observations reinforce their findings, in addition demonstrating that baseline CTC numbers are predictive of 13-week responses. Most previous studies have used AJCC 7th edition and, by doing so, OPC patients were staged as comparatively advanced compared to more recent studies where AJCC 8th edition was used. Consequently, care should be taken when comparing previous data in studies employing AJCC 7th edition staging for p16 positive OPC patients.

We also found a strong correlation between CTCs and other tumour characteristics. Mean CTC counts at baseline were associated with tumour stage (AJCC 8th Ed) and lymph-node involvement in the whole cohort of patients. In p16 positive OPC patients, the mean CTC counts were significantly higher in N stage 2–3 compared to N stage 0–1 (2.38 vs. 1.21) cases. Moreover, mean CTC counts were significantly higher in stage III compared to stage I and II patients (2.29 vs. 1.45). Similar patterns were observed for other HNCs where the mean CTC count was higher in N stage 2–3 compared to N stage 0–1 patients (2.0 vs. 2.33) and stage III compared to stage I and II patients (3.25 vs. 0.4). However, the mean CTC count in stage IVa and IVb patients was 1.25, which was lower than that in stage III patients but higher than that in stage I and II patients. Several previous HNC studies, including the aforementioned meta-analysis by Xun et al., also suggested that CTC counts may increase as the tumour burden increases [24, 50, 51]. A few other studies have, however, reported contradictory findings [52, 53].

Considering that EMT promotes tumour cell migration, we also investigated the EMT status of CTCs based on CSV expression. We found that CSV expression was significantly higher in patients with incomplete responses compared to patients with complete responses. Putative prognostic implications of EMT characterization in CTCs have been reported across several cancer types [54]. A study conducted on hepatocellular carcinoma reported that the presence of high CTC counts with a higher fraction of mesenchymal CTCs relates to high local and distant metastatic rates [55]. Furthermore, a study conducted by Batth et al. [56] on neuroblastoma patients, indicated that patients with CSV-positive CTCs exhibit high recurrence rates. Similar indications have been reported in HNC by TADA et al. [29] where they found that CTCs with hybrid epithelial and mesenchymal characteristics form major fractions of CTCs in HNC patients with poor treatment outcomes. These findings, together with our current findings, emphasise that incorporating secondary EMT (CSV) scoring may improve the prognostic utility of CTCs in HNC.

Our study has a few limitations. Even though, we have initially recruited 135 HNC patients to the study, 16 of them did not undergo 13 weeks PET-CT scans due to various reasons. Moreover, fewer than expected patients were non-responders. As this is a prospective study, where treatment naïve HNC patients were recruited at diagnosis, we could not control for the exact number of complete and incomplete responders and, consequently, the analysis involving response status has less power than expected. We presented confidence intervals for all parameter estimates allowing evaluation of the precision of our results. However, further research will be required to confirm and validate our results. In the initial stage of the study, 36 patients were categorized as having equivocal PET-CT results, but were subsequently categorized as complete or incomplete responders based on follow-up PET-CT scans and MDT consensus data. This report is part of a long-term study and, thus, HNC patients considered in the study are being followed up further to investigate the long-term prognostic utility of CTC detection. Investigation of CSV expression in CTCs was conducted as a pilot project and, hence, its sample size is small (n = 10).

## Conclusions

From our data we conclude that baseline CTC numbers in HNC patients are significantly correlated with treatment outcome at 13 weeks post-treatment as determined by PET-CT. Patients without baseline CTCs are anticipated to show complete responses at 13-weeks and CTCs can be used as additional prognostic indicators when stratifying patients for risk. Furthermore, we found that CSV expression in CTCs was significantly higher in HNC patients with adverse clinical outcomes and, thus, the incorporation of CSV scores may further improve the prognostic utility of CTCs in HNC. Detection of CTCs at baseline can be considered as a potential risk stratification tool in HNC.

## Electronic supplementary material

Below is the link to the electronic supplementary material.

Supplementary Material 1

Supplementary Material 2

Supplementary Material 3

## Data Availability

The datasets generated during and/or analysed during the current study are available from the corresponding author upon reasonable request.
