# Supplementary figures and images for: Application of circulating tumour cells to predict response to treatment in head and neck cancer
Source: Cell Oncol (Dordr). 2022 Jun 23;45(4):543–55. doi: 10.1007/s13402-022-00681-w (PMC9219366; doi:10.1007/s13402-022-00681-w)

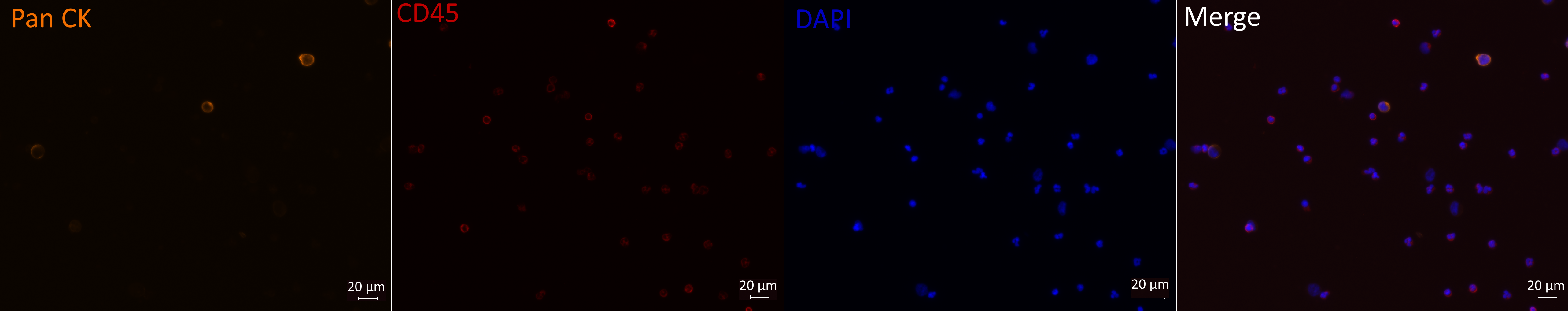

Supplement: Supplementary file 1 — Supplementary Material 1 [file 13402_2022_681_MOESM1_ESM.png]

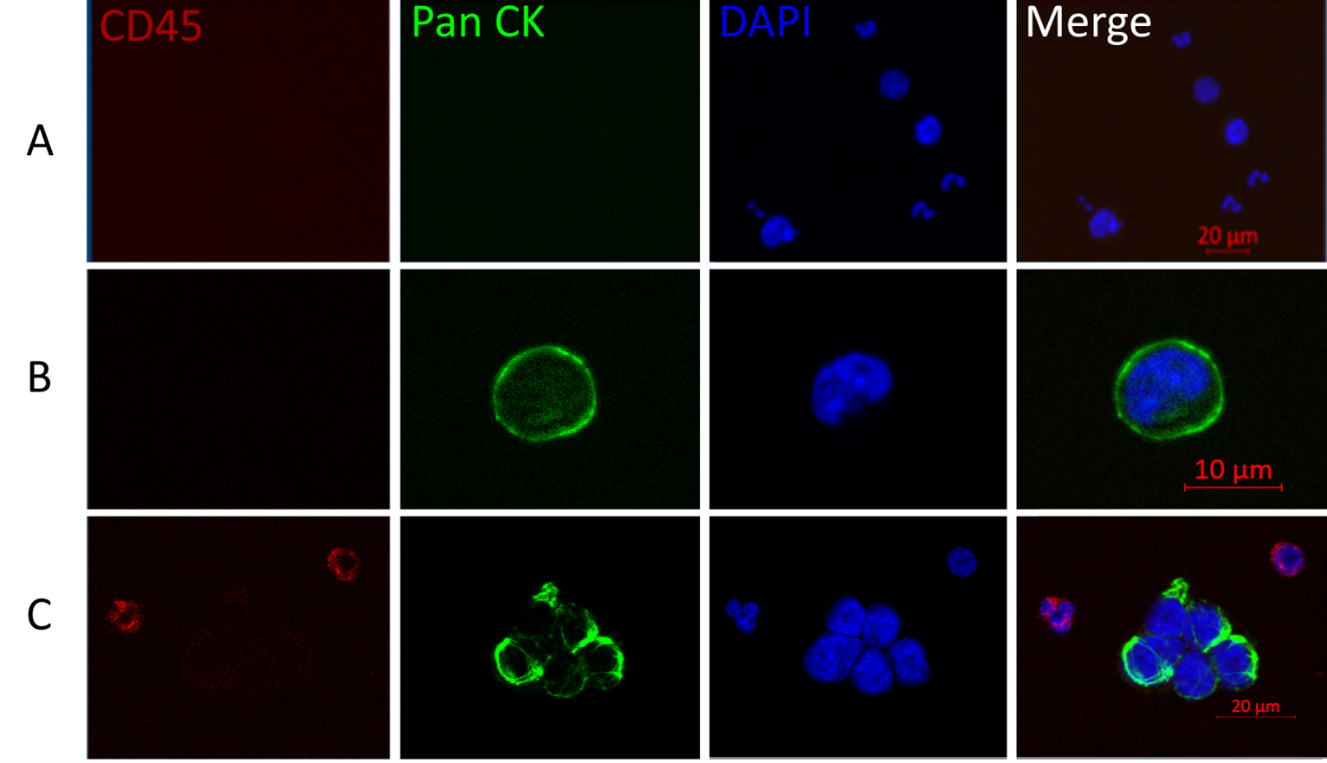

Supplement: Supplementary file 2 — Supplementary Material 2 [file 13402_2022_681_MOESM2_ESM.png]

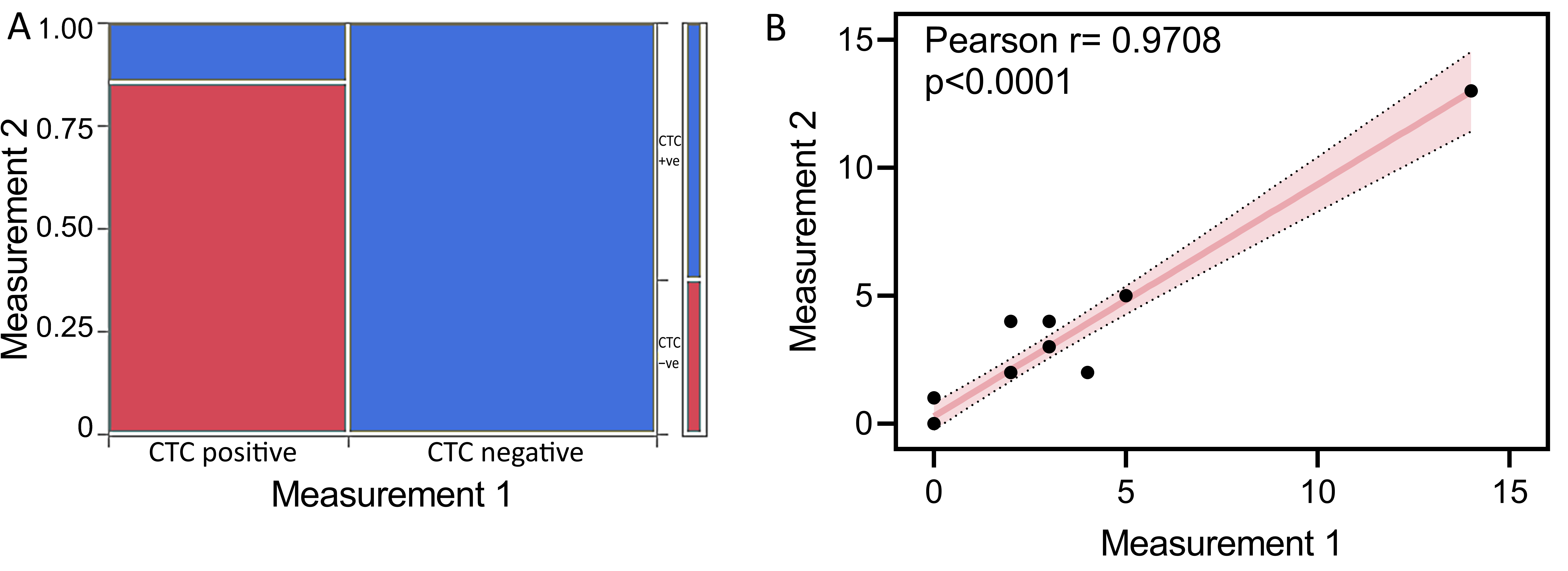

Supplement: Supplementary file 3 — Supplementary Material 3 [file 13402_2022_681_MOESM3_ESM.png]
